# Psychological predictors of cesarean delivery on maternal request in upper middle-income settings and the mitigating role of prenatal psychological education: a multicenter longitudinal study

**DOI:** 10.3389/fmed.2025.1683360

**Published:** 2026-01-06

**Authors:** Tianjiao Liu, Dongqiong Luo, Yanhong Li, Juan Li, Xin Li, Lu Zhang, Lan Huang, Hongyan Ma, Biao Huang, Aijie Xie

**Affiliations:** 1 Department of Obstetrics and Gynecology, Chengdu Women's and Children's Central Hospital, School of Medicine, University of Electronic Science and Technology of China, Chengdu, China; 2 Department of Obstetrics and Gynecology, Chengdu Xinjin District Maternal and Child Health Care Hospital, Chengdu, China; 3 Clinical Laboratory, The People's Hospital of Lincang, Lincang, China; 4 Department of Obstetrics and Gynecology, Chengdu Jintang County Maternal and Child Health Hospital, Chengdu, Sichuan, China; 5 Department of Obstetrics and Gynecology, Women and Children's Hospital of Chongqing Medical University, Chongqing, China; 6 NHC Key Laboratory of Birth Defects and Reproductive Health, Chongqing, China

**Keywords:** cesarean section, maternal anxiety, maternal depression, pregnancy mental health, prenatal psychological education

## Abstract

**Objective:**

This study aimed to investigate, in an upper middle–income country context, the impact of maternal psychological status and prenatal psychological education on the cesarean delivery on maternal request among primiparous women.

**Methods:**

This prospective longitudinal study included 948 primiparous women who delivered at five hospitals between June 2023 and June 2024. Women with medical indications for cesarean section, multiparity, or incomplete data were excluded. Maternal anxiety and depression were assessed in the first, second, and third trimesters using the Self-Rating Anxiety Scale and Self-Rating Depression Scale. Childbirth fear was evaluated in the third trimester using the Wijma Delivery Expectancy/Experience Questionnaire (W-DEQ). Multivariate logistic regression was used to examine factors associated with cesarean delivery on maternal request.

**Results:**

Among the 948 participants, 543 (57.3%) had vaginal deliveries, while 405 (42.7%) underwent cesarean delivery on maternal request. Third-trimester anxiety, depression, and W-DEQ scores were significantly higher in the cesarean group (*P* < 0.001 for all). Multivariate analysis revealed that conception via assisted reproductive technology (ART; OR = 2.12, 95% CI: 1.17–3.43), the presence of pregnancy-related illness (OR = 1.43, 95% CI: 1.09–2.08), and higher W-DEQ scores (OR = 1.08, 95% CI: 1.02–1.16) were independent risk factors for cesarean delivery on maternal request. Receipt of prenatal psychological education was associated with a 35% reduction in this risk (OR = 0.65, 95% CI: 0.38–0.91).

**Conclusion:**

Maternal psychological distress and fear of childbirth are significant contributors to the decision for cesarean delivery on maternal request among primiparous women. In an upper middle–income country context, prenatal psychological education may serve as an effective intervention to reduce childbirth fear and promote informed, evidence-based delivery decisions. Integrating routine mental health screening and targeted education into antenatal care could help curb the rising trend of unnecessary cesarean deliveries.

## Background

The global rise in cesarean section (CS) rates has become a pressing concern in obstetrics, particularly for cesarean delivery on maternal request (CDMR) (1, 2). While cesarean delivery can be life-saving under appropriate clinical circumstances, elective CDMR has been associated with increased risks of maternal complications, neonatal respiratory morbidity, and prolonged postpartum recovery, as well as greater healthcare costs and resource burden (3).

In recent years, China has experienced a persistently elevated and gradually increasing cesarean section rate (4). Recent multi-center estimates place the overall cesarean rate at approximately 39% nationally (5). Internationally, upper middle income countries report markedly higher incidences of CDMR than high income countries, and some jurisdictions exceed overall cesarean rates of 50%−60% (6, 7); for example, Cyprus reports 57%, Turkey reported 62.2% of births by cesarean in 2023, and Puerto Rico reported 50.5% in 2022 (8, 9). A growing body of evidence suggests that non-medical factors—including maternal psychological status and childbirth-related fear—play a significant role in influencing the decision for CDMR (7, 10). Primiparous women, lacking prior childbirth experience, are particularly vulnerable to childbirth anxiety and negative expectations, which may lead them to request a cesarean delivery as a perceived means of reducing uncertainty, pain, or risk (11, 12).

Anxiety and depressive symptoms during pregnancy, especially in the third trimester, have been shown to influence maternal attitudes toward childbirth and preferences for delivery mode (13, 14). Psychological distress can amplify fear of labor pain, loss of control, or complications, thereby increasing the likelihood of CDMR. Fear of childbirth encompasses multiple psychological dimensions, including anticipated pain, perceived loss of control, and anxiety about maternal or neonatal complications. Among primiparous women, limited prior experience may amplify negative expectations and reduce confidence in coping with labor, which can shift preferences toward cesarean delivery even in the absence of medical indications (15). Cognitive appraisal and affective responses during late pregnancy are particularly salient, as they shape perceived risks and decision making at term. These mechanisms provide the rationale for measuring childbirth fear using validated tools and for examining its association with CDMR (16, 17).

Meanwhile, prenatal psychological education—an intervention aimed at improving emotional preparedness and childbirth confidence—has demonstrated potential benefits in reducing anxiety and correcting misconceptions about childbirth (18). However, empirical data on its effectiveness in preventing elective cesarean delivery among primiparas remain limited, particularly in the context of contemporary Chinese maternal healthcare systems.

This study aims to investigate the impact of psychological factors and prenatal psychological education on the likelihood of elective CDMR among primiparous women. By analyzing longitudinal changes in maternal mental health during pregnancy and evaluating the role of childbirth expectations, this research seeks to identify modifiable psychological determinants and inform targeted interventions to promote evidence-based delivery decisions.

## Methods

### Study design and participants

**Figure 1:**
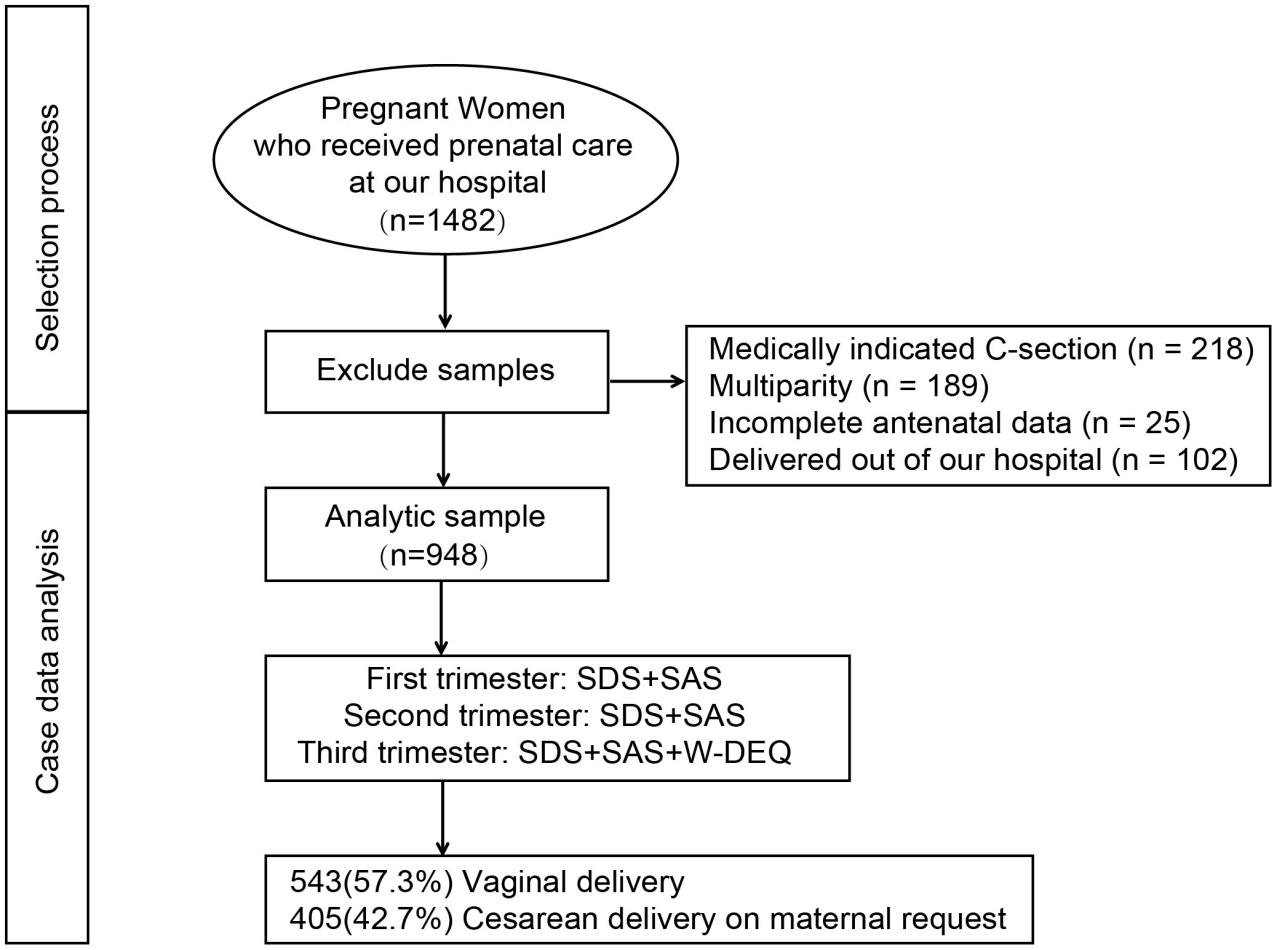
The selection process for this study.

This prospective longitudinal study was conducted at Chengdu Xinjin District Maternal and Child Healthe Care Hospital and other tertiary-level hospitals between June 2023 and June 2024. The study protocol was approved by the Ethics Committee of Chengdu Women's and Children's Central Hospital. Eligible participants were primiparous women aged 18–40 years with singleton pregnancies at ≥37 weeks of gestation, who asked CDMR and provided written informed consent. Women were excluded if they had severe obstetric complications (e.g., severe preeclampsia, placental abruption), major psychiatric disorders or cognitive impairments that would prevent completion of questionnaires, or a history of uterine surgery, including previous cesarean section or myomectomy. Eligible primiparous women were consecutively approached at their first antenatal visit and followed at each trimester clinic. Screening, exclusions, enrolment, and analysis populations are shown in Figure 1.

### Data collection

Sociodemographic and clinical data were obtained through medical records and structured interviews, including maternal age, height, pre-pregnancy weight, occupation, gravidity, parity, mode of conception, and surgical history. Pre-pregnancy lifestyle factors (e.g., smoking, alcohol use) and pregnancy complications (e.g., gestational diabetes mellitus, gestational hypertension, hypothyroidism, and intrahepatic cholestasis of pregnancy) were documented. Maternal psychological status was assessed using standardized instruments during each trimester. The Self-Rating Anxiety Scale (SAS) and Self-Rating Depression Scale (SDS) were administered in the first, second, and third trimesters to evaluate anxiety and depressive symptoms (19). The Wijma Delivery Expectancy/Experience Questionnaire (W-DEQ) was used during the third trimester to assess fear of childbirth (20). All questionnaires were administered by trained personnel following standardized protocols. Delivery mode (vaginal delivery or CDMR) was recorded postnatally.

CDMR was defined as a cesarean delivery performed in the absence of predefined maternal or fetal indications recorded in the chart. All indications were abstracted from standardized delivery records and independently reviewed by two senior obstetricians.

SDS: The SDS consists of 20 items, evenly divided between positive and negative statements. Scores are summed and divided by 80 to generate an index. Depression severity was categorized as follows: < 0.50 = no depression; ≥0.50 = depression.

SAS: The SAS comprises 20 items assessing subjective anxiety. The total raw score is multiplied by 1.25 to yield a standardized score, categorized as: < 50 = no anxiety; ≥0.50 = anxiety.

DEQ: The W-DEQ includes 33 items, with 16 assessing positive and 17 assessing negative emotions. Negative items are reverse-coded, yielding a total score ranging from 0 to 165. A score ≥85 is commonly considered indicative of clinically significant childbirth fear. The W-DEQ, a validated tool for assessing childbirth fear and expectations, has been widely used to quantify these psychological factors and predict delivery decisions.

Validated Chinese versions of the SAS, SDS, and W-DEQ were used (21); in our cohort, Cronbach's α was 0.87 for SAS, 0.84 for SDS, and 0.91 for W-DEQ. Prespecified cut-offs were SAS ≥ 50, SDS index ≥ 0.50, and W-DEQ ≥ 85.

In this study, pregnancy-induced illness was defined as physician-diagnosed conditions arising *de novo* during gestation and documented in the medical record—for example, gestational diabetes mellitus and new-onset hypothyroidism—that, in themselves, do not constitute indications for cesarean delivery. All diagnoses followed national or international guideline criteria and were coded as binary variables.

### Antenatal psychological intervention

Given ethical and risk considerations, most hospitals in China permit patient-requested cesarean delivery, and the decision is made in accordance with the woman's preference after counseling. For women requesting cesarean due to fear of childbirth, clinicians routinely recommend antenatal psychological intervention; this is encouraged but optional rather than mandatory. A standardized, hospital-based program was delivered between 24 and 34 weeks by trained perinatal psychologists or midwives in small groups, comprising two 60–90 min sessions on psychoeducation about labor, cognitive restructuring for fear, breathing and relaxation skills, partner involvement, and birth planning; printed materials and short videos were provided, attendance was recorded in the electronic medical record, and exposure was coded as receipt of at least one structured session with a sensitivity analysis using completion of two sessions.

### Statistical analysis

Data were analyzed using SPSS version 25.0 (IBM Corp., Armonk, NY, USA). Categorical variables were compared using the Chi-square test or Fisher's exact test, as appropriate. Continuous variables were expressed as means ± standard deviations and compared using independent-sample t-tests or Welch's t-tests in cases of unequal variance. A multivariate linear regression model was used to identify factors associated with W-DEQ scores in the third trimester. Binary logistic regression analysis was performed to explore associations between perinatal factors and CDMR. Covariates were entered into the multivariable logistic model if they showed association at *p* < 0.05 in univariate tests or were identified *a priori* from the literature as potential confounders. All tests were two-tailed, and *P* values < 0.05 were considered statistically significant.

## Results

The participant selection process is depicted in Figure 1. A total of 1,482 pregnant women who received prenatal care at our hospital during the study period were initially assessed. Based on the inclusion and exclusion criteria, 218 cases of medically indicated cesarean section, 189 multiparous women, 25 women with incomplete antenatal records, and 102 women who delivered outside the hospital were excluded. The final analytic sample comprised 948 primiparous women. Among the included participants, 543 (57.3%) underwent vaginal delivery, while 405 (42.7%) had CDMR. The mean maternal age was 29.58 ± 3.99 years, with a mean gestational age at delivery of 39.48 ± 1.66 weeks. The mean birthweight of the newborns was 2,950.31 ± 581.12 grams, and the mean birth length was 49.41 ± 2.45 cm. A total of 31 women (3.3%) conceived through assisted reproductive technology (Table 1).

**Table 1:** Description of the maternal and neonatal characteristics.

**Variables**	**Total**
Mothers	948
Maternal age (year)	29.583 ± 0.99
Pre-pregnancy BMI (kg/m^2^)	22.203 ± 0.64
**Mode of conception**	
Natural conception	917 (96.7%)
Assisted reproductive technology	31 (3.3%)
Gestational weight gain (kg)	12.843 ± 0.29
Gestational age (week)	39.481 ± 0.66
Smoking	28 (3.0%)
Alcohol consumption history	43 (4.5%)
Pregnancy-induced illness	237 (25.0%)
Gravidity	1 (1)
D&C artificial abortion	0 (1)
**Mode of delivery**	
Vaginal delivery	543 (57.3%)
Cesarean delivery on maternal request	405 (42.7%)
Infants	948
Gender (male)	484 (51.1%)
Birthweight (g)	2,950.315 ± 81.12
Birth length (cm)	49.412 ± 0.45

BMI, body mass index; D&C, Dilation and Curettage.

Compared to the vaginal delivery group, women who underwent CDMR had significantly higher pre-pregnancy BMI (22.82 ± 3.85 vs. 21.73 ± 3.48, *P* < 0.001), a higher incidence of pregnancy-induced illnesses (33.1% vs. 19.0%, *P* < 0.001), and a lower rate of receiving prenatal psychological education (42.5% vs. 54.7%, *P* < 0.001). In late pregnancy, they also showed significantly higher scores on the Anxiety Scale (54.74 ± 8.73 vs. 50.24 ± 7.85, *P* < 0.001), Depression Scale (41.16 ± 5.44 vs. 37.73 ± 4.81, *P* < 0.001), and the Wijma Delivery Expectancy/Experience Questionnaire (83.77 ± 10.95 vs. 78.16 ± 9.13, *P* < 0.001). No significant differences were observed between groups regarding maternal age, gestational age at delivery, birthweight, or birth length (Table 2).

**Table 2:** Description of the maternal and neonatal characteristics by mode of delivery.

**Variables**	**Vaginal delivery**	**CDMR**	***P*-value**
Mothers	*N* = 543	*N* = 405	
Age (year)	29.503 ± 0.93	29.694 ± 0.08	0.471^a^
Pre-pregnancy BMI (kg/m^2^)	21.733 ± 0.48	22.823 ± 0.85	< 0.001^a^
**Mode of conception**			0.034^b^
Natural conception	531 (97.8%)	386 (95.3%)	
Assisted reproductive technology	12 (2.2%)	19 (4.7%)	
Gestational weight gain (kg)	12.683 ± 0.19	13.063 ± 0.42	0.082^a^
Gestational age (week)	39.421 ± 0.74	39.571 ± 0.55	0.162^a^
Smoking	16 (2.9%)	12 (3.0%)	0.988^b^
Alcohol consumption history	25 (4.6%)	18 (4.4%)	0.907^b^
Pregnancy-induced illness	103 (19.0%)	134 (33.1%)	< 0.001^b^
Gravidity	1 (1)	2 (1)	0.339^c^
D&C artificial abortion	0 (1)	1 (1)	0.124^c^
Prenatal psychological education	297 (54.7%)	172 (42.5%)	< 0.001^b^
**Psychological status in late pregnancy**			
Anxiety scale score	50.247 ± 0.85	54.748 ± 0.73	< 0.001^a^
Depression scale score	37.734 ± 0.81	41.165 ± 0.44	< 0.001^a^
Wijma-delivery expectancy/experience questionnaire	78.169 ± 0.13	83.771 ± 0.95	< 0.001^a^
Infants	*N* = 543	*N* = 405	
Gender (male)	287 (52.9%)	197 (48.6%)	0.199^b^
Birthweight (g)	2,923.755 ± 78.30	2,985.915 ± 84.88	0.104^a^
Birth length (cm)	49.372 ± 0.36	49.462 ± 0.57	0.581^a^

CS, cesarean section; BMI, body mass index; D&C, dilation and curettage.

^a^Average and standard deviation. Student's t-Test.

^b^Number (percentage). Chi-squared Test.

^c^Median (interquartile range). Kruskal-Wallis Test.

**Figure 2:**
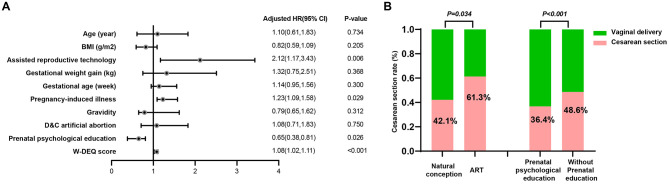
The impact of participant characteristics on cesarean delivery on maternal request. **(A)** After adjustment for maternal age, pre-pregnancy BMI, gestational weight gain, gravidity, and history of D and C, non-medically indicated cesarean section was more likely with ART conception (aOR 2.12, 95% CI 1.17–3.43, *P* = 0.006), pregnancy-induced illness (aOR 1.23, 95% CI 1.09–1.58, *P* = 0.029), and higher W-DEQ scores (per point aOR 1.08, 95% CI 1.02–1.11, *P* < 0.001), and less likely with prenatal psychological education (aOR 0.65, 95% CI: 0.38–0.81, *P* = 0.026). **(B)** Subgroup analyses were concordant: CDMR was more frequent after ART vs. natural conception (61.3% vs. 42.1%, *P* = 0.034) and among women without vs. with prenatal psychological education (48.6% vs. 36.4%, *P* < 0.001).

Binary logistic regression was used to further investigate risk factors for CDMR. After adjusting for potential covariates including maternal age, pre-pregnancy BMI, gestational weight gain, gravidity, and history of D&C artificial abortion, the binary logistic regression analysis revealed that several factors were independently associated with an increased risk of CDMR (Figure 2A). Specifically, conception via assisted reproductive technology (ART) was associated with a significantly higher risk (adjusted OR = 2.12, 95% CI: 1.17–3.43, *P* = 0.006), as was the presence of pregnancy-induced illness (adjusted OR = 1.23, 95% CI: 1.09–1.58, *P* = 0.029). Higher scores on the W-DEQ were also positively associated with elective cesarean delivery (adjusted OR = 1.08, 95% CI: 1.02–1.11, *P* < 0.001), indicating that each 10-point increase in W-DEQ score corresponded to an 80% increase in the risk of CDMR. Conversely, receipt of prenatal psychological education was significantly associated with a reduced risk of CDMR (adjusted OR = 0.65, 95% CI: 0.38–0.81, *P* = 0.026), reflecting a 35% risk reduction.

Subgroup analysis further supported these findings. As shown in Figure 2B, the rate of CDMR was significantly higher among women who conceived via ART compared to those with natural conception (61.3% vs. 42.1%, *P* = 0.034). Additionally, women who did not receive prenatal psychological education had a significantly higher rate of non-indicated cesarean delivery compared to those who received such education (48.6% vs. 36.4%, *P* < 0.001).

As shown in Table 3, both anxiety and depression symptoms exhibited a statistically significant upward trend across trimesters. The mean anxiety score increased from 47.10 ± 7.31 in the first trimester to 49.64 ± 7.94 in the second trimester, reaching 52.16 ± 8.21 in the third trimester (*P* < 0.001). Correspondingly, the proportion of pregnant women with clinically significant anxiety rose from 43.4% (*n* = 412) in the first trimester to 49.4% (*n* = 469) in the second trimester, and further to 58.9% (*n* = 558) in the third trimester (*P* = 0.030). Similarly, depression scores showed a progressive increase from 37.77 ± 4.62 in the first trimester to 38.43 ± 5.05 in the second trimester and 39.20 ± 5.56 in the third trimester (*P* = 0.012). The proportion of women meeting the threshold for depressive symptoms increased from 41.7% (*n* = 395) to 44.3% (*n* = 420), and further to 48.1% (*n* = 456) across the same periods (*P* = 0.013). These findings suggest a significant deterioration in maternal psychological well-being as pregnancy progresses, particularly in the third trimester.

**Table 3:** Longitudinal trajectory of maternal psychological well-being throughout pregnancy.

**Variables**	**First trimester**	**Second trimester**	**Third trimester**	***P*-value**
Anxiety scale score	47.107 ± 0.31	49.647 ± 0.94	52.168 ± 0.21	< 0.001^a^
Proportion with anxiety	412 (43.4%)	469 (49.4%)	558 (58.9%)	0.030^b^
Depression scale score	37.774 ± 0.62	38.435 ± 0.05	39.205 ± 0.56	0.012^a^
Proportion with depression	395 (41.7%)	420 (44.3%)	456 (48.1%)	0.013^b^

^a^Average and standard deviation. One-way Analysis of Variance.

^b^Number (percentage). Chi-squared Test.

As shown in Table 4, multiple linear regression analysis was conducted to identify perinatal factors associated with third-trimester scores on the W-DEQ. The model explained 29.0% of the variance in W-DEQ scores (R^2^ = 0.290). Conception via assisted reproductive technology (β = 3.37, 95% CI: 1.86–4.88, *P* < 0.001), higher pre-pregnancy BMI (β = 1.65, 95% CI: 1.22–2.08, *P* < 0.001), the presence of pregnancy-induced illness (β = 1.30, 95% CI: 0.85–1.75, *P* < 0.001), higher third-trimester anxiety scores (β = 2.19, 95% CI: 1.55–2.83, *P* < 0.001), and depression scores (β = 1.87, 95% CI: 0.72–3.02, *P* = 0.001) were all positively associated with higher W-DEQ scores, indicating more negative or fearful childbirth expectations. Conversely, receipt of prenatal psychological education was significantly associated with lower W-DEQ scores (β = −4.56, 95% CI: −6.73 to −2.39, *P* < 0.001), suggesting a beneficial effect in reducing fear or negative expectations toward childbirth.

**Table 4:** Multivariate analysis of perinatal factors associated with Wijma-delivery expectancy/experience questionaire scores in the third trimester.

**Variables**	**Beta**	**95% CI**	***P*-value**	**VIF**
**R**^2^ = **0.290**				
Age (year)	1.17	(−0.85, 3.19)	0.256	1.22
BMI (g/m^2^)	1.65	(1.22, 2.08)	< 0.001	1.20
Assisted reproductive technology	3.37	(1.86, 4.88)	< 0.001	1.09
Gestational weight gain (kg)	1.26	(−0.67, 3.19)	0.201	1.27
Gestational age (week)	1.13	(−0.94, 3.2)	0.285	1.32
Pregnancy-induced illness	1.30	(0.85, 1.75)	< 0.001	1.18
Gravidity	0.78	(−0.43, 1.99)	0.206	1.27
D&C artificial abortion	1.07	(−0.67, 2.81)	0.228	1.12
Anxiety scale score	2.19	(1.55, 2.83)	< 0.001	1.34
Depression scale score	1.87	(0.72, 3.02)	0.001	1.40
Prenatal psychological education	−4.56	(−6.73, −2.39)	< 0.001	1.46

BMI, body mass index.

## Discussion

This study explored the influence of maternal psychological factors and prenatal psychological education on the likelihood of CDMR among primiparous women. The findings underscore the complex interplay between psychological well-being, childbirth expectations, and delivery mode, revealing that elevated anxiety, depressive symptoms, and childbirth-related fear in the third trimester are associated with an increased risk of non-medically indicated cesarean delivery. Importantly, prenatal psychological education emerged as a potentially protective factor, significantly reducing the probability of CDMR.

The observed association between maternal psychological distress and CDMR is consistent with prior research, which has identified anxiety and fear of childbirth as key psychological drivers of delivery preferences, particularly among primiparas (12, 13, 22). High W-DEQ scores—indicative of increased fear of childbirth—may reflect a lack of confidence in vaginal delivery, heightened perception of labor pain, or mistrust in one's ability to cope with delivery-related uncertainty, all of which may influence women to proactively request cesarean delivery even in the absence of clinical indications (23, 24).

Notably, our findings contribute to a growing body of literature suggesting that psychological education during pregnancy may mitigate these concerns. Structured antenatal psychological interventions may enhance maternal self-efficacy, reduce catastrophic thinking, and normalize the emotional responses associated with childbirth preparation (25, 26). By improving cognitive appraisal and emotional regulation, psychological education may serve as a low-cost, scalable intervention to counteract the rising trend of unnecessary cesarean sections in China and other high-burden countries.

The high rate of CDMR observed in this cohort −42.7%—further highlights the urgency of addressing non-clinical determinants of delivery mode. Despite national policy efforts, China's cesarean section rate remains substantially above the World Health Organization's recommended threshold of 10%−15% (27), with considerable regional variation. This suggests the influence of institutional practices, provider preferences, and sociocultural norms, in addition to maternal psychological status. Among primiparous women, fear of pain, lack of childbirth experience, and insufficient support during labor may further compound the preference for surgical delivery (28, 29). Although the CDMR group showed a 33.1% prevalence of pregnancy-induced illness in univariable comparisons, in this study pregnancy-induced illness explicitly excluded conditions that require cesarean delivery; therefore, these diagnoses do not independently mandate cesarean, and these women remained eligible for vaginal birth.

Another noteworthy finding is the psychological vulnerability of women who conceived via assisted reproductive technologies (ART). These women were significantly more likely to report higher childbirth fear and undergo CDMR. This may reflect a tendency toward risk-avoidance among ART pregnancies, potentially due to heightened concern for fetal well-being or perceived fragility of the pregnancy (30, 31). Tailored psychological support for this subgroup may be warranted.

Routine trimester-specific mental health screening using validated Chinese versions of SAS and SDS, together with third-trimester W-DEQ, could be incorporated into antenatal care pathways. A stepped-care model can be implemented in which brief, structured antenatal psychoeducation is delivered by midwives or perinatal psychologists, with referral to higher-intensity services when indicated.

This study has several strengths, including its prospective design, use of validated psychological assessment tools across trimesters, and adjustment for a comprehensive set of clinical covariates. Because delivery mode was captured through the hospital electronic record for all included participants, outcome-level loss to follow-up was minimal. However, certain limitations must be acknowledged. First, psychological assessments were based on self-reported measures, which may be subject to response bias. Second, the generalizability of the findings may be limited by the single-center design and regional context. Given our observational design, findings indicate associations rather than causation. Multicenter randomized controlled trials are needed to test whether structured prenatal psychological education reduces W-DEQ scores and the proportion of CDMRs compared with usual care.

## Conclusion

In conclusion, this study highlights the pivotal role of maternal psychological well-being and prenatal psychological education in shaping delivery decisions. Interventions aimed at identifying and addressing childbirth-related anxiety and fear—particularly in the third trimester—may contribute to reducing unnecessary cesarean sections. Integrating routine psychological screening and targeted education into prenatal care may be a crucial strategy to promote safe, informed, and evidence-based childbirth choices among primiparous women.

## Data Availability

The raw data supporting the conclusions of this article will be made available by the authors, without undue reservation.
